# Association of systemic immune-inflammation index with diabetic kidney disease in patients with type 2 diabetes: a cross-sectional study in Chinese population

**DOI:** 10.3389/fendo.2023.1307692

**Published:** 2024-01-04

**Authors:** Pijun Yan, Yuxia Yang, Xing Zhang, Yi Zhang, Jia Li, Zujiao Wu, Xiaofang Dan, Xian Wu, Xiping Chen, Shengxi Li, Yong Xu, Qin Wan

**Affiliations:** ^1^ Department of Endocrinology and Metabolism, The Affiliated Hospital of Southwest Medical University, Luzhou, China; ^2^ Metabolic Vascular Disease Key Laboratory of Sichuan Province, Luzhou, China; ^3^ Sichuan Clinical Research Center for Diabetes and Metabolism, Luzhou, China; ^4^ Sichuan Clinical Research Center for Nephropathy, Luzhou, China; ^5^ Cardiovascular and Metabolic Diseases Key Laboratory of Luzhou, Luzhou, China; ^6^ Department of Clinical Nutrition, Chengdu Eighth People’s Hospital (Geriatric Hospital of Chengdu Medical College), Chengdu, China; ^7^ Clinical Medical College, Southwest Medical University, Luzhou, China

**Keywords:** systemic immune-inflammation index, diabetic kidney disease, distinct phenotypes, Chinese population, biomarker

## Abstract

**Objective:**

Systemic immune-inflammation index (SII), a novel inflammatory marker, has been reported to be associated with diabetic kidney disease (DKD) in the U.S., however, such a close relationship with DKD in other countries, including China, has not been never determined. We aimed to explore the association between SII and DKD in Chinese population.

**Methods:**

A total of 1922 hospitalized patients with type 2 diabetes mellitus (T2DM) included in this cross-sectional study were divided into three groups based on estimated glomerular filtration rate (eGFR) and urinary albumin-to-creatinine ratio (ACR): non-DKD group, DKD stages 1–2 Alb group, and DKD-non-Alb+DKD stage 3 Alb group. The possible association of SII with DKD was investigated by correlation and multivariate logistic regression analysis, and receiver-operating characteristic (ROC) curves analysis.

**Results:**

Moving from the non-DKD group to the DKD-non-Alb+DKD stage 3 Alb group, SII level was gradually increased (*P* for trend <0.01). Partial correlation analysis revealed that SII was positively associated with urinary ACR and prevalence of DKD, and negatively with eGFR (all *P*<0.01). Multivariate logistic regression analysis showed that SII remained independently significantly associated with the presence of DKD after adjustment for all confounding factors [(odds ratio (OR), 2.735; 95% confidence interval (CI), 1.840-4.063; *P* < 0.01)]. Moreover, compared with subjects in the lowest quartile of SII (Q1), the fully adjusted OR for presence of DKD was 1.060 (95% CI 0.773-1.455) in Q2, 1.167 (95% CI 0.995-1.368) in Q3, 1.266 (95% CI 1.129-1.420) in the highest quartile (Q4) (*P* for trend <0.01). Similar results were observed in presence of DKD stages 1–2 Alb or presence of DKD-non- Alb+DKD stage 3 Alb among SII quartiles. Last, the analysis of ROC curves revealed that the best cutoff values for SII to predict DKD, Alb DKD stages 1- 2, and DKD-non-Alb+ DKD stage 3 Alb were 609.85 (sensitivity: 48.3%; specificity: 72.8%), 601.71 (sensitivity: 43.9%; specificity: 72.3%), and 589.27 (sensitivity: 61.1%; specificity: 71.1%), respectively.

**Conclusion:**

Higher SII is independently associated with an increased risk of the presence and severity of DKD, and SII might be a promising biomarker for DKD and its distinct phenotypes in Chinese population.

## Introduction

Diabetic kidney disease (DKD) is one of the most common and severe chronic diabetic microvascular complications that is clinically characterized by a gradual decline in renal function, with or without proteinuria (1), affecting approximately 20%–40% of people with type 2 diabetes mellitus (T2DM) (2, 3). Currently, DKD has become the leading cause of chronic kidney disease (CKD) and end-stage renal disease (ESRD) requiring dialysis or transplantation (2, 4), and also is a major risk factor for cardiovascular events and mortality (1, 2), resulting in a significant burden on the public health and economic systems of countries throughout the world. However, DKD is asymptomatic in early stages and its treatment option is limited (3), and thus a complete cure for DKD is an unmet medical need that urgently requires the discovery of novel reliable biomarkers that can guide the early identification and further treatment of DKD.

The systemic immune- inflammation index (SII), a novel inflammatory biomarker integrating three different cells, including neutrophil, lymphocyte, and platelet, was originally used to estimate the prognosis of patients with hepatocellular carcinoma by Hu et al. in 2014 (4, 5), and then has been developed to predict the prognosis in other malignant tumors types, such as colorectal cancer, cervical cancer, lung cancer, esophageal cancer, oropharyngeal cancers, epithelial ovarian cancer, papillary thyroid carcinoma, and melanoma (4, 6, 7). Now, SII is thought to better and more objectively reflect the state of inflammation and immune balance in the body compared with white blood cells and its subtypes (neutrophil and lymphocyte) as well as the neutrophil-to- lymphocyte ratio and platelet-to-lymphocyte (5, 8, 9), and predict the prognosis of certain autoimmune disorders, such as autoimmune encephalitis, systemic lupus erythematosus, and adult-onset Still’s disease, and inflammatory diseases, such as acute pancreatitis, ulcerative colitis, and chronic obstructive pulmonary disease (4, 10–15). Recently, growing evidence suggests that SII may be associated with metabolic disorder and its components, such as central obesity, nonalcoholic fatty liver disease, metabolic syndrome, dyslipidemia, and hypertension (15–19), all of which have been reported to be involved in the development and progression of DKD (20). Furthermore, it has been demonstrated that elevated SII levels are associated with subclinical atherosclerosis, and can efficiently predict the development, prognosis and clinical outcomes of various atherosclerotic macrovascular diseases, such as acute coronary syndrome, myocardial infarction, coronary artery disease (CAD), heart failure (HF), stroke, peripheral arterial disease (PAD), and diabetic foot infections (4, 8–10, 13, 15, 16, 18, 21–27), all of which were closely related to DKD (28, 29). Given that chronic inflammation and metabolic disorder are involved in the pathogenesis of DKD, and that atherosclerotic macro- and microvascular diseases share multiple common pathogenetic pathways and risk factors, it is reasonable to hypothesize that T2DM individuals with high SII would have a high risk for DKD. Indeed, only a cross-sectional study from the National Health and Nutrition Examination Survey (NHANES) between 2011 and 2018 in the U.S. showed that higher SII level was associated with DKD among 3937 T2DM patients (4). However, it could not be determined whether there is a graded association between SII quartiles and risk of DKD and its distinct phenotypes, and whether SII could predict the presence of distinct phenotypes of DKD. Moreover, no study thus far has evaluated the associations between SII and DKD in China, where early onset of type 2 diabetes was reported and patients with T2DM have a higher prevalence of DKD, albuminuria, and a faster deterioration of renal function than their Caucasian counterparts in the U.S.

Therefore, to fill this gap in knowledge, we conducted a cross-sectional study to explore the association between SII and DKD and distinct phenotypes of DKD in Chinese adults with T2DM.

## Methods

### Study population

A total of 3514 adult inpatients with T2DM who had visited the hospital’s department of endocrinology between August 2012 and September 2015 were initially selected. T2DM was defined as fasting blood glucose (FBG) ≥7.0 mmol/L, 2-h plasma glucose level on their oral glucose tolerance test (OGTT) ≥11.1 mmol/L, self-reported diagnosis of diabetes by a physician, or use of antidiabetic medications (30). All participants underwent the face-to-face questionnaire interview, systematic physical examinations, blood and urine sample collection, and diabetic complications examinations. The exclusion criteria were as follows: (1) type 1 diabetes mellitus, gestational diabetes, and other specific types of diabetes, acute diabetic complications; (2) non-diabetic kidney disease (such as membranous and IgA nephropathy, systemic lupus erythematosus, ANCA-associated vasculitis), recent history ofdialysis for acute kidney failure or a kidney transplant; (3) liver and gallbladder diseases; (4) inflammatory diseases, infectious disease, presence of stressful conditions (recent surgery, trauma); (5) symptomatic chronic heart failure, acute cardiovascular events (such as hospitalization for heart failure, myocardial infarction, andstroke within three months), severe respiratory failure; (6) autoimmune disease, immunosuppressant, use of systemic glucocorticoid; (7) thromboembolic disease, hematological system diseases; (8) malignant tumours; (9) pregnancy or lactation; and (10) missing baseline data and without available information. Subsequently, 1922 participants (975 men and 947 women) were included in the analysis.

This study was in accordance with the principles of the Declaration of Helsinki, and was approved by the Ethics Committee of the Affiliated Hospital of Southwest Medical University. All patients completed the signing of informed consent form before being enrolled.

### General clinical and biochemical measurements

A face-to-face interview was carried out by well-trained interviewers to collect information on demographic data (sex, age), lifestyle factors (smoking status, alcohol consumption, etc.), personal medical history [coronary heart disease (CHD), stroke, and symptomatic PAD], medication history, and family history (diabetes, hypertension, etc.) with a standard questionnaire. Body weight and height were measured following standardized procedures, and body mass index (BMI) was calculated as weight divided by height squared (30, 31). The patients’ systolic and diastolic blood pressure (SBP, DBP) were measured three times using a mercury sphygmomanometer while the subject in a sitting position for at least 5 min, and the mean value was recorded (21, 31). Mean arterial pressure (MAP) and pulse pressure (PP) were calculated: PP = SBP − DBP and MAP = DBP + (1/3) PP (32).

Biochemical indicators, including FBG, 2h postprandial blood glucose (PBG), total cholesterol (TC), triglyceride (TG), high density lipoprotein cholesterol (HDL-C), low-density lipoprotein cholesterol (LDL-C), apolipoprotein A (apoA), apolipoprotein B (apoB), alanine aminotransferase (ALT), aspartate aminotransferase (AST), total bilirubin (TBIL), serum creatinine (Cr), glycated hemoglobin A1C (HbA1c), neutrophil count, lymphocyte count, hemoglobin (Hb) and platelet (PLT) count, were assayed through venous blood samples obtained in the morning after an overnight fast (≥ 8h). ApoB/A is the ratio between the concentrations of apoB and apoA. The SII was calculated as platelet × neutrophil/lymphocyte counts (4, 5, 9, 17, 21). The glycemic exposure (GE) index was calculated using the following equation: GE index = (HbA1c) ^1/2^x (duration of DM in years) ^1/8^ (33). Metabolic score for insulin resistance (METS-IR) was calculated as (ln [(2 × FBG) + TG)] × BMI)/(ln [HDL-C]) (FBG, TG, and HDL-C levels expressed as mg/dL and BMI as kg/m^2^ in the equation) (34).

### Assessment and diagnostic criteria of DKD

The estimated glomerular filtration rate (eGFR; mL/min/1.73 m^2^) was calculated using the Chronic Kidney Disease Epidemiology Collaboration (CKD-EPI) equation that includes age, sex, and race (4, 17, 31). Urinary albumin-to-creatinine ratio (ACR) was measured by random spot urine for three times with at least two positive results out of three tests (31). DKD was diagnosed with low eGFR (eGFR <60 mL/min/1.73 m^2^), albuminuria (urinary ACR ≥30 mg/g), or both in T2DM patients (4, 31, 35).

### Definitions of clinical variables

Patients were considered to have overweight/obesity when BMI ≥ 24 kg/m^2^ (36). Hypertension was defined as SBP ≥ 140 mmHg and/or DBP ≥ 90 mmHg and/or presence of anti-hypertensive drug treatment (8, 18, 36). Glycaemic control was assessed in terms of the HbA1c level and poor glycaemic control was defined as HbA1c ≥7% according to the American Diabetes Association (37). Dyslipidaemia was defined as either incident abnormal lipid laboratory results (comprised of TC >200 mg/dL, TG >150 mg/dL, LDL-C >130 mg/dL, or HDL-C <40 mg/dL) or incident lipid-lowering medications prescriptions (consisting of prescription of statins, bile acid resins, and fibrates) (18, 38). In accordance to the AHA/ACC 2018 cholesterol management guidelines, atherosclerotic cardiovascular disease (ASCVD) consisted of CHD (myocardial infarction, angina, or coronary revascularization), stroke (hemorrhagic and ischemic stroke), and symptomatic PAD (i.e., a history of PAD with claudication, gangrene or ulceration, peripheral artery revascularization, or major amputation secondary to PAD) (39, 40). DR was determined by using fundus photography (Canon Inc., Kanagawa, Japan), which was performed by an ophthalmologist (31).

### Statistical analysis

All statistical analyses were performed using the Statistical Package for Social Sciences (SPSS) (version 20.0; IBM, Chicago, IL). Continuous variables were expressed as mean ± standard deviation (SD), and compared by Student’s t test, Mann-Whitney U, one-way analysis of variance (ANOVA), and Kruskal-Wallis H tests, while categorical variables were described using number (percentage), and compared by chi-squared test. The correlations between SII and other variables with significant differences were determined using Spearman’s correlation and partial correlation analysis. The univariate and multivariable logistic regression analyses were conducted to investigate the association of SII and other variables with the risk of presence of DKD, reporting the data as odds ratio (OR) with a 95% confidence interval (CI). All participants were divided into DKD group and non-DKD (eGFR ≥60 mL/min/1.73 m^2^ and UACR < 30 mg/g), and then DKD group was categorized into three subgroups: DKD-non-Alb (eGFR < 60 mL/min/1.73 m^2^ and urinary ACR < 30 mg/g) subgroup; Alb DKD stage 3 (DKD stage 3 Alb, eGFR < 60 mL/min/1.73 m^2^ and urinary ACR ≥ 30 mg/g) subgroup; and Alb DKD stages 1- 2 (DKD stages 1–2 Alb, eGFR ≥60 mL/min/1.73 m2 and urinary ACR ≥ 30 mg/g) subgroup (35, 41). DKD-non-Alb subgroup and DKD stage 3 Alb subgroup were merged into a group called DKD-non-Alb+DKD stage 3 Alb subgroup due to limited sample sizes of DKD-non-Alb subgroup (n=56). A multivariate logistic regression model was used to estimate ORs and 95% CIs for the association of SII as a continuous variable with DKD and different stages of DKD. Then, SII was classified into four quartiles, and the associations between SII quartiles and DKD and different stages of DKD was investigated, with the lowest quartile as the reference group. Last, the receiver operating characteristic (ROC) curves were constructed to evaluate the sensitivity and specificity of SII in predicting DKD, DKD stages 1–2 Alb, and DKD-non-Alb+DKD stage 3 Alb, and area under the curve (AUC) was estimated. A two-sided p-value<0.05 was deemed to be of statistical significance.

## Results

### Clinical and laboratory characteristics

**Figure 1:**
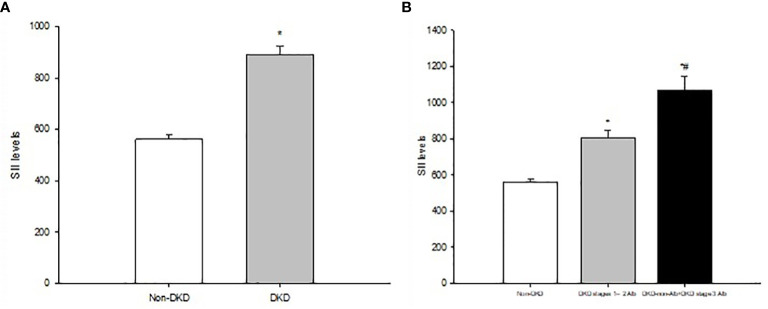
**(A)** Systemic immune-inflammatory index (SII) levels in T2DM patients with non-DKD and DKD. **(B)** SII levels in T2DMpatients with non-DVD Alb DKD stages 1-2, and DKD-non-Alb+DKD stage 3 Alb. Data are expressed as means ± SD. *P<0.01 vs. non-DKD, *P<0.01 vs. Alb DKD stages 1-2.

A total of 1922 participants were enrolled, of whom 1063 (55.31%), 724 (37.67%), and 339 (17.64%) patients had DKD, DKD stages 1–2 Alb, and DKD-non-Alb+DKD stage 3 Alb, respectively. **Table 1** and **Figure 1** displayed the SII levels and other clinical and laboratory characteristics of the 3 evaluated groups. Among three groups, differences with statistical significance were observed in age, duration of diabetes, family history of diabetes, SBP, MAP, PP, HDL-C, apoA, apoB/A, FBG, PBG, HbA1c, GE index, neutrophil and lymphocyte count, SII, ALT, TBIL, Hb, serum Cr, eGFR, urinary ACR, prevalence of poor glycaemic, hypertension, dyslipidaemia, DR, and ASCVD (*P*<0.01 or *P*<0.05). The subgroup with eGFR <60 mL/min/1.73 m^2^ (DKD-non-Alb and DKD stage 3 Alb) tended to be older, with a longer duration of diabetes, a higher SBP, PP, apoB/A, FBG, PBG, HbA1c, neutrophil count, SII, serum Cr, urinary ACR, prevalence of poor glycaemic, hypertension, and ASCVD, and a lower lymphocyte count, ALT, TBIL, Hb, and eGFR compared with the DKD stages 1–2 Alb and non-DKD subgroups (*P*<0.01 or *P*<0.05). Of note, SII levels in T2DM patients with DKD (DKD stages 1–2 Alb, and DKD-non-Alb and DKD stage 3 Alb) were significantly higher than those in T2DM patients with non-DKD (*P*<0.01; **Figure 1**). The DKD-non-Alb and DKD stage 3 Alb subgroup was less likely to have a family history of diabetes, tended to have a higher MAP, GE index, prevalence of dyslipidaemia and DR, and a lower apoA compared with the non-DKD subgroup (*P*<0.01 or *P*<0.05). Compared with the non-DKD subgroup, the DKD stages 1–2 Alb subgroup had a lower HDL-C (*P*<0.05). **Supplementary Table 1** displayed the SII levels and other clinical characteristics in T2DM patients with Non-DKD and DKD. Compared with T2DM patients with non-DKD, those with DKD had significantly longer diabetic duration, higher age, SBP, MAP, PP, TG, apoB/A, METS-IR, GE index, neutrophil count, SII, serum Cr, urinary ACR, prevalence of hypertension, dyslipidaemia, DR, and ASCVD, and lower HDL-C, apoA, lymphocyte count, ALT, TBIL, Hb, and eGFR (*P*<0.01 or *P*<0.05).

**Table 1:** Baseline characteristics of study participants stratified by DKD phenotype.

Characteristic	Non-DKD	DKD stages 1–2 Alb	DKD-non-Alb+DKD stage 3 Alb	*P*
	(n =859) (44.69%)	(n =724) (37.67%)	(n =339) (17.64%)	
Male, n (%)	457 (53.20)	353 (48.76)	165 (48.67)	0.150
Age (years)	57.84 ± 10.93	61.18 ± 11.20^**^	67.06 ± 9.56^**##^	0.000
BMI (kg/m^2^)	24.26 ± 3.46	24.35 ± 4.22	24.65 ± 3.63	0.276
Duration of diabetes (years)	6.41 ± 5.75	8.77 ± 6.34^**^	11.08 ± 7.25^**##^	0.000
Family history of diabetes, n (%)	241 (28.06)	192 (26.52)	67 (19.76)^*^	0.012
Family history of hypertension, n (%)	82 (9.55)	76 (10.50)	32 (9.44)	0.782
Smoking, n (%)	180 (20.95)	164 (22.65)	63 (18.58)	0.311
Drinking, n (%)	149 (17.35)	119 (16.44)	49 (14.45)	0.478
SBP (mmHg)	127.73 ± 19.73	135.87 ± 21.81^**^	143.37 ± 24.65^**##^	0.000
DBP (mmHg)	71.76 ± 11.97	73.15 ± 12.56	72.15 ± 14.64	0.069
MAP (mmHg)	90.42 ± 12.70	94.06 ± 13.42^**^	95.89 ± 16.00^**^	0.000
PP (mmHg)	55.98 ± 16.99	62.75 ± 19.42^**^	71.23 ± 20.07^**##^	0.000
TC (mmol/L)	4.75 ± 1.22	4.77 ± 1.45	4.82 ± 1.63	0.681
TG (mmol/L)	2.21 ± 2.40	2.47 ± 3.01	2.14 ± 1.48	0.061
HDL-C (mmol/L)	1.18 ± 0.35	1.14 ± 0.38^*^	1.17 ± 0.48	0.010
LDL-C (mmol/L)	2.74 ± 0.92	2.69 ± 1.02	2.81 ± 1.26	0.276
ApoA (g/L)	1.36 ± 0.35	1.26 ± 0.32^**^	1.26 ± 0.33^**^	0.000
ApoB (g/L)	0.88 ± 0.25	0.91 ± 0.35	0.92 ± 0.34	0.627
ApoB/A	0.69 ± 0.25	0.76 ± 0.36^**^	0.77 ± 0.35^**##^	0.000
FBG (mmol/L)	10.63 ± 4.77	11.13 ± 5.31	10.01 ± 6.24^**##^	0.000
PBG (mmol/L)	15.89 ± 5.20	16.49 ± 5.21	14.90 ± 4.53^**##^	0.000
HbA1c (%)	9.32 ± 2.44	9.62 ± 2.57	8.55 ± 2.39^**##^	0.000
GE index	3.47 ± 0.78	3.80 ± 0.77^**^	3.72 ± 0.75^**^	0.000
METS-IR	40.72 ± 8.64	42.06 ± 10.58	41.38 ± 8.81	0.105
Neutrophil (*10^9^/L)	4.18 ± 1.89	4.89 ± 2.51^**^	5.63 ± 3.16^**##^	0.000
Lymphocyte (*10^9^/L)	1.71 ± 0.67	1.61 ± 0.61^**^	1.38 ± 0.56^**##^	0.000
PLT (×10^9^/L)	194.47 ± 64.27	208.58 ± 87.29	202.96 ± 83.58	0.118
SII	563.07 ± 18.43	807.95 ± 36.19^**^	1070.36 ± 74.42^**##^	0.000
ALT (U/L)	25.37 ± 24.25	23.81 ± 27.38^**^	19.16 ± 14.48^**##^	0.000
AST (U/L)	22.38 ± 16.68	23.68 ± 23.60	22.47 ± 26.04	0.435
TBIL (μmol/L)	13.21 ± 5.77	11.87 ± 5.98^**^	9.80 ± 4.62^**##^	0.000
Hb (g/L)	134.82 ± 17.12	124.99 ± 19.32^**^	108.41 ± 22.18^**##^	0.000
Serum Cr (μmol/L)	60.52 ± 15.85	65.26 ± 18.52^**^	194.81 ± 146.56^**##^	0.000
eGFR (mL/min/1.73 m^2^)	101.25 ± 16.60	94.44 ± 20.15^**^	37.10 ± 16.27^**##^	0.000
Urinary ACR (mg/g)	13.34 ± 7.59	37.93 ± 31.00^**^	153.64 ± 11.30^**#^	0.000
Overweight/obesity, n (%)	411 (47.85)	348 (48.07)	154 (45.43)	0.321
Poor glycaemic control, n (%)	707 (82.31)	616 (85.08)	234 (69.03)^**##^	0.000
Hypertension, n (%)	371 (43.19)	451 (62.29)^**^	283 (83.48)^**##^	0.000
Dyslipidaemia, n (%)	578 (67.29)	512 (70.72)	257 (75.81)^*^	0.016
DR, n (%)	69 (8.03)	116 (16.02)^**^	67 (19.76)^**^	0.000
ASCVD, n (%)	211 (24.56)	259 (35.77)^**^	171 (50.44)^**##^	0.000

Data are mean ± SD. SD, standard deviation; DKD, Diabetic kidney disease; BMI, body mass index; SBP, systolic blood pressure; DBP, diastolic blood pressure; MAP, mean arterial pressure; PP, pulse pressure; TC, total cholesterol; TG, triglyceride; HDL-C, high-density lipoprotein cholesterol; LDL-C, low-density lipoprotein cholesterol; apoA, apolipoprotein A; apoB, apolipoprotein B; apoB/A, apolipoprotein B-to-apolipoprotein A ratio; FBG, fasting blood glucose; PBG, 2 hour postprandial blood glucose; HbA1c, glycated hemoglobin A1c; GE index, glycemic exposure index; METS-IR, metabolic score for insulin resistance; PLT, platelet; SII, systemic immune-infammation index; ALT, alanine aminotransferase; AST, aspartate aminotransferase; TBIL, total bilirubin; Hb, hemoglobin; Cr, creatinine; eGFR, estimated glomerular filtration rate; ACR, albumin- to-creatinine ratio; DR, diabetic retinopathy; ASCVD, atherosclerotic cardiovascular disease. vs. non-DKD: ^*^P< 0.05, ^**^P< 0.01, vs. Alb DKD stages 1- 2: ^#^P< 0.05, ^##^P< 0.01.

### The relationships between SII and DKD- related risk factors

We used Spearman correlation analysis to test the correlation between SII and cardiometabolic risk factors. The results showed that SII was positively associated with age, SBP, PP, apoB/A, FBG, PBG, HbA1c, GE index, neutrophil count, PLT count, serum Cr, urinary ACR, prevalence of low eGFR, albuminuria, DKD, poor glycaemic, and hypertension, and negatively with BMI, drinking, TC, TG, apoA, METS-IR, lymphocyte count, ALT, AST, TBIL, Hb, eGFR, and prevalence of overweight/obesity (*P*<0.01 or *P*<0.05; **Table 2**). Partial correlation analysis controlling for sex, age, BMI, and duration of diabetes demonstrated that SII was positively associated with apoB/A, FBG, PBG, HbA1c, GE index, METS-IR, neutrophil count, PLT count, serum Cr, urinary ACR, prevalence of low eGFR, albuminuria, DKD, and poor glycaemic, and inversely correlated with TC, HDL-C, lymphocyte count, TBIL, Hb, and eGFR (*P*<0.01 or *P*<0.05; **Table 2**).

**Table 2:** The relationships between SII and DKD- related risk factors.

Variables	*r*	*P*-value	Adjusted *r*	Adjusted *P*-value
Age	0.100	0.000		
Sex	0.041	0.071		
Duration of diabetes	0.020	0.381		
BMI	-0.127	0.000		
Family history of diabetes	-0.020	0.381	0.007	0.772
Family history of hypertension	-0.008	0.726	0.009	0.721
Smoking	-0.013	0.571	-0.009	0.729
Drinking	-0.056	0.014	-0.031	0.229
SBP	0.056	0.015	-0.008	0.746
DBP	-0.035	0.124	-0.023	0.365
MAP	0.011	0.619	-0.006	0.816
PP	0.085	0.000	0.017	0.496
TC	-0.098	0.000	-0.060	0.019
TG	-0.127	0.000	-0.009	0.737
HDL-C	0.002	0.948	-0.109	0.000
LDL-C	-0.004	0.846	-0.011	0.653
ApoA	-0.157	0.000	-0.253	0.000
ApoB	0.005	0.824	0.018	0.475
ApoB/A	0.116	0.000	0.226	0.000
FBG	0.113	0.000	0.165	0.000
PBG	0.103	0.000	0.105	0.000
HbA1c	0.087	0.000	0.119	0.000
GE index	0.084	0.000	0.097	0.000
METS-IR	-0.096	0.000	0.125	0.000
Neutrophil	0.722	0.000	0.705	0.000
Lymphocyte	-0.468	0.000	-0.329	0.000
PLT	0.548	0.000	0.447	0.000
ALT	-0.204	0.000	-0.023	0.366
AST	-0.211	0.000	0.020	0.432
TBIL	-0.191	0.000	-0.075	0.003
Hb	-0.221	0.000	-0.225	0.000
Serum Cr	0.165	0.000	0.104	0.000
eGFR	-0.202	0.000	-0.132	0.000
Urinary ACR	0.226	0.000	0.104	0.000
Low eGFR	0.199	0.000	0.133	0.000
Albuminuria	0.204	0.000	0.157	0.000
DKD	0.220	0.000	0.166	0.000
DR	0.014	0.547	-0.032	0.216
Overweight/obesity	-0.119	0.000	-0.034	0.157
Poor glycaemic control	0.071	0.002	0.091	0.000
Dyslipidaemia	-0.020	0.394	0.004	0.872
Hypertension	0.090	0.000	0.022	0.398
ASCVD	0.035	0.121	0.035	0.168

### Univariate and multivariate logistic analysis of factors associated with DKD

**Table 3** showed univariable and multivariable analyses of factors associated with DKD. On univariable analysis, age, duration of diabetes, GE index, hypertension, dyslipidaemia, METS-IR, apoB, apoA, apoB/A, ALT, TBIL, Hb, SII, PP, DR, and ASCVD were significantly associated with DKD (*P*<0.01 or *P*<0.05). SII remained independently significantly associated with an increased risk of DKD on multivariable analysis (OR = 2.735, 95% CI 1.840-4.063; *P*<0.01).

**Table 3:** Univariate and multivariate logistic analysis of factors associated with DKD.

Variables	Univariate analysis			Multivariate analysis		
	B	OR (95%CI)	*P*-value	B	OR (95%CI)	*P*-value
Female sex	0.179	1.196 (0.999-1.432)	0.051	-0.435	0.648 (0.501-0.837)	0.001
Age	0.043	1.044 (1.035-1.053)	0.000			
BMI	0.012	1.012 (0.987-1.037)	0.338			
Duration of diabetes	0.081	1.084 (1.067-1.101)	0.000	0.035	1.036 (1.010-1.063)	0.007
Family history of diabetes	-0.191	0.826 (0.673-1.013)	0.067			
Family history of hypertension	0.069	1.072 (0.792-1.450)	0.654			
Smoking	0.024	1.024 (0.822-1.277)	0.831			
Drinking	-0.112	0.894 (0.703-1.139)	0.365			
HbA1c	-0.006	0.994 (0.959-1.030)	0.726			
GE index	0.501	1.650 (1.463-1.861)	0.000	0.197	1.218 (1.011-1.467)	0.038
Hypertension	1.077	2.935 (2.433-3.540)	0.000	0.590	1.804 (1.386-2.347)	0.000
Dyslipidaemia	0.245	1.277 (1.045-1.561)	0.017			
METS-IR	0.013	1.013 (1.003-1.024)	0.013	0.023	1.023 (1.009-1.038)	0.001
apoA	-0.912	0.402 (0.298-0.541)	0.000	-1.003	0.367 (0.175-0.770)	0.008
apoB	0.320	1.377 (1.014-1.868)	0.040	1.298	3.661 (1.329-10.086)	0.012
apoB/A	0.908	2.479 (1.772-3.468)	0.000			
ALT	-0.006	0.994 (0.990-0.999)	0.009			
AST	0.002	1.002 (0.998-1.007)	0.353			
TBIL	-0.065	0.937 (0.920-0.954)	0.000	-0.021	0.979 (0.959-0.999)	0.036
Hb	-0.041	0.960 (0.955-0.965)	0.000	-0.036	0.965 (0.957-0.972)	0.000
SII	1.525	4.597 (3.361-6.288)	0.000	1.006	2.735 (1.840-4.063)	0.000
PP	0.027	1.028 (1.023-1.033)	0.000			
DR	0.867	2.381 (1.776-3.192)	0.000	0.573	1.773 (1.247-2.523)	0.001
ASCVD	0.735	2.086 (1.712-2.543)	0.000	0.312	1.366 (1.051-1.774)	0.020

B is the standardized coefficient and measures the influence of each variables on DKD; OR is the odds ratio and refers to the risk of DKD.

### Adjusted ORs and 95% CIs for DKD, DKD stages 1–2 Alb, and DKD-non-Alb+DKD stage 3 Alb according to SII quartiles

Multivariate logistic regression analysis showed that the SII, whether considered as a categorical or continuous variable, remained significant after adjusting for confounders (**Table 4**). As a continuous variable, SII was associated with a 4.2-fold increased risk of DKD in the partially adjusted regression model 1, and a 2.8-fold increased risk of DKD in fully adjusted model 4. As a categorical variable, compared with subjects in the lowest quartile (Q1), the partially adjusted OR for DKD was 1.134 (95% CI 0.859-1.496) in the second quartile (Q2), 1.233 (95% CI 1.072-1.417) in the third quartile (Q3), and 1.407 (95% CI 1.276-1.553) in the highest quartile (Q4), respectively. The increased risk of DKD from Q1 to Q4 was statistically significant (*P* for trend<0.01). A similar pattern was observed in fully adjusted model 4 (Q2: OR=1.060, 95% CI 0.773-1.455; Q3: OR=1.167, 95% CI 0.995-1.368; Q4: OR=1.266, 95% CI 1.129-1.420; *P* for trend <0.01). Moreover, we further divided DKD group into two subgroups: DKD stages 1–2 Alb subgroup and DKD-non-Alb+DKD stage 3 Alb subgroup, and studied the associations between the SII and different stages of DKD. The result revealed that SII was associated with a 2.3-fold increased risk of DKD stages 1–2 Alb (*P* < 0.01), and there was a graded association with DKD stages 1–2 Alb and increase in SII quartiles infully adjusted model 4 (*P* for trend < 0.01). Participants in the Q4 of SII had a significantly higher risk of DKD stages 1–2 Alb compared with those in the Q1 (OR = 1.211, 95% CI 1.073-1.366). Similarly, SII was associated with a 4.2-fold increased risk of DKD-non-Alb+DKD stage 3 Alb (*P* < 0.01), and the increased risk of DKD-non-Alb+DKD stage 3 Alb from Q1 to Q4 was also statistically significant in fully adjusted model 4 (*P* for trend < 0.01).

**Table 4:** Adjusted ORs and 95% CI for DKD according to SII quartiles.

SII	OR (95% CI)			
	Model 1	Model 2	Model 3	Model 4
DKD				
Continuous	4.234 (3.002-5.973)^**^	3.389 (2.328-4.933)^**^	2.681 (1.793-4.010)^**^	2.780 (1.855-4.166)^**^
Categories				
Q1	1 (reference)	1 (reference)	1 (reference)	1 (reference)
Q2	1.134 (0.859-1.496)	1.086 (0.809-1.459)	1.076 (0.788-1.470)	1.060 (0.773-1.455)
Q3	1.233 (1.072-1.417)^**^	1.179 (1.014-1.371)^*^	1.161 (0.991-1.359)	1.167 (0.995-1.368)
Q4	1.407(1.276-1.553)^**^	1.342 (1.206-1.494)^**^	1.263 (1.128-1.414)^**^	1.266 (1.129-1.420)^**^
*P* for trend	0.000	0.000	0.000	0.000
DKD stages 1–2 Alb				
Continuous	3.391 (2.351-4.891)^**^	2.642 (1.766-3.952)^**^	2.197 (1.438-3.357)^**^	2.289 (1.494-3.506)^**^
Categories				
Q1	1 (reference)	1 (reference)	1 (reference)	1 (reference)
Q2	1.062 (0.793-1.423)	1.013 (0.743-1.382)	1.028 (0.746-1.419)	1.023 (0.738-1.417)
Q3	1.138 (0.980-1.320)	1.095 (0.933-1.286)	1.089 (0.923-1.285)	1.095 (0.927-1.293)
Q4	1.326 (1.195-1.471)^**^	1.265 (1.129-1.417)^**^	1.205 (1.070-1.357)^**^	1.211 (1.073-1.366)^**^
*P* for trend	0.000	0.000	0.002	0.002
DKD-non-Alb+DKD stage 3 Alb				
Continuous	9.323 (5.393-16.116)^**^	7.273 (3.982-13.284)^**^	3.966 (2.003-7.854)^**^	4.220 (2.118-8.409)^**^
Categories				
Q1	1 (reference)	1 (reference)	1 (reference)	1 (reference)
Q2	1.606 (0.965-2.674)	1.664 (0.957-2.895)	1.514 (0.745-3.081)	1.457 (0.709-2.993)
Q3	1.648 (1.293-2.101)^**^	1.560 (1.194-2.037)^**^	1.537 (1.124-2.101)^**^	1.541 (1.125-2.110)^**^
Q4	1.684 (1.435-1.977)^**^	1.582 (1.324-1.892)^**^	1.414 (1.148-1.740)^**^	1.439 (1.164-1.779)^**^
*P* for trend	0.000	0.000	0.001	0.000

Logistic regression analysis with DKD group and each DKD subgroup in contradistinction to non-DKD group to investigate the association between SII quartiles and DKD stages 1–2 Alb or DKD-non-Alb+DKD stage 3 Alb. Data are expressed as OR (95% CI), unless stated otherwise. OR, odds ratio; CI, confidence interval.

Model 1 adjusted for sex, age, BMI, duration of diabetes, family history of diabetes, family history of hypertension, smoking, drinking;

Model 2 adjusted for factors listed in Model 1 plus HbA1c, GE, hypertension, dyslipidaemia, apoA, apoB, apoB/A, METS-IR;

Model 3 adjusted for factors listed in Model 2 plus ALT, AST, TBIL, Hb;

Model 4 adjusted for factors listed in Model 3 plus PP, DR, ASCVD;

^*^P < 0.05.

^**^P < 0.01.

### Predictive value of SII in screening for the presence of DKD in T2DM patients

**Figure 2:**
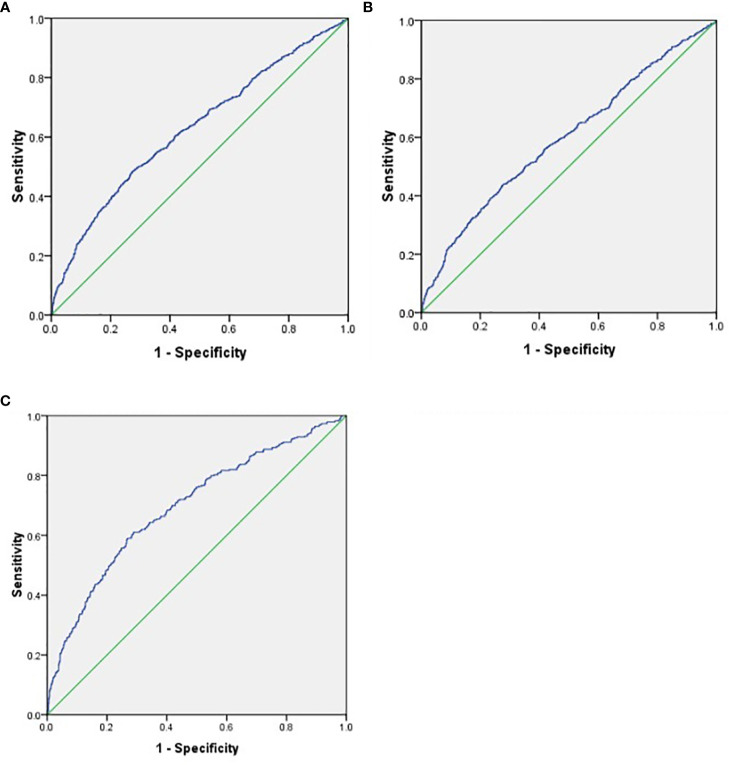
**(A)** ROC analysis of systemic immune-inflammatory index (SII) to indicate DKD. AUC = 0_627; 95% CI: 0.603–0.652; *P*<0.01; identified SII cutoff value = 609.85; Youden index = 0.211; sensitivity: 48.3%; specificity: 72.8%. **(B)** ROC analysis of indicate Alb DVD stages 1-2. AUC = 0.596; Cl: 0.568–0.624; *P* <0.01; identified SII cutoff value = 601.71; Youden index = 0.162; sensitivity: 43.9%; specificity: 72.3%. **(C)** ROC analysis of SII to indicate DKD-non-Alb+DKD stage 3 Alb. AUC = 0.695; 95% CI: 0.661–0.729; *P*<0.01; identified SII cutoff value = 589.27; Youden index = 0.322; sensitivity: 61.1%; specificity: 71.1%.

To further explore the predictive value of SII for DKD and different stages of DKD, the ROC curve analysis was performed. As shown in **Figure 2**, the best cut-off value of SII was 609.85 for predicting DKD (sensitivity: 48.3%; specificity: 72.8%; and AUC: 0.627; **Figure 2A**), 601.71 (sensitivity: 43.9%; specificity: 72.3%; and AUC: 0.596; **Figure 2B**) for predicting DKD stages 1–2 Alb, and 589.27 (sensitivity: 61.1%; specificity: 71.1%; and AUC: 0.695; **Figure 2C**) for predicting DKD-non-Alb+DKD stage 3 Alb.

## Discussion

The current study was the first to investigate the associations of SII, as an indicator of inflammatory marker, with the presence of DKD and its distinct phenotypes in Chinese population with T2DM. We discovered that moving from the non-DKD group to the DKD-non-Alb+DKD stage 3 Alb group, SII level was gradually increased, and SII remained independently significantly associated with the presence of DKD after adjustment for confounding factors. Additionally, the risk of DKD and its distinct phenotypes increased progressively across SII quartiles. Last, the ROC curve analysis revealed that SII could predict DKD and its distinct phenotypes. Our finding suggested that SII may be a useful biomarker of DKD and its distinct phenotypes, and high SII may be associated with an increased risk of DKD and its distinct phenotypes in Chinese adults with T2DM.

As mentioned earlier, SII is a relatively novel inflammation biomarker based on peripheral lymphocyte, neutrophil, and platelet counts in clinical applications, which largely reflects three pathways of inflammatory response, thrombus formation and adaptive immune response in the host (5, 22, 27, 42). In the last decade, SII has been demonstrated to predict the prognosis and clinical outcomes of various malignant tumors types, certain inflammatory and autoimmune diseases, atherosclerosis, cardio-cerebrovascular diseases (5, 8–16, 22, 23, 25, 27). Recently, some studies have reported the association between SII and kidney diseases with varying epidemiological methods and target populations (5, 43–46). More recently, SII has been reported to be associated with metabolic disorder, such as metabolic syndrome, obesity, hepatic steatosis, dyslipidemia and hypertension, and diabetic vascular complications, such as diabetic retinopathy, diabetic macular edema, peripheral arterial disease, and diabetic foot infections (15–19, 22, 24–27, 47, 48). Considering the role of SII in the development and progression of autoimmune and inflammatory diseases and kidney diseases, and the close relationship between DKD and metabolic disorder, diabetic vascular complications, it seems appropriate that SII may be associated with DKD, and high SII may be an early signal for being at risk for DKD. In the present study, we found that SII levels were significantly. higher in T2DM patients with DKD than those with non-DKD, and SII levels were further increased in T2DM patients with DKD-non-Alb and DKD stage 3 Alb compared to those with DKD stages 1–2 Alb, suggesting that SII might be related to DKD and its distinct phenotypes. Moreover, SII was positively associated with serum Cr, urinary ACR, an early hallmark of DKD, prevalence of low eGFR, albuminuria, and DKD, and inversely with eGFR. In addition, the multivariate logistic regression analysis showed that SII had an independent positive correlation with DKD in T2DM patients, and ROC curve analysis revealed that the SII could effectively predict the presence of DKD. These findings were consistent with a previous cross-sectional study from the NHANES in the U.S. that reported that a high SII level was associated with increased likelihood of DKD among 3937 T2DM patients (4). All the above findings suggested that a higher SII was significantly associated with an increased risk of having DKD, which supported the hypothesis that SII may serve as a promising biomarker for identifying patients at a higher risk of DKD. However, it could not be determined whether there is a graded association between SII quartiles and risk of presence of DKD and its distinct phenotypes, and whether SII could predict the presence of distinct phenotypes of DKD. Our study filled this gap and demonstrated for the first time that the risk of prevalence of DKD increased progressively across SII quartiles, and participants in the highest quartile was at a significantly increased risk of prevalent DKD compared to those in the lowest quartile, even after adjusting for potential confounding factors. A similar pattern was observed regarding the association of SII quartiles with distinct phenotypes of DKD, including DKD stages 1–2 Alb, and DKD-non-Alb+DKD stage 3 Alb. Besides, SII was found to effectively predict the presence of distinct phenotypes of DKD, especially DKD-non-Alb+DKD stage 3 Alb, with good sensitivity and specificity. Such discoveries suggested that high SII could be linked to an increased risk of DKD and its distinct phenotypes, and SII might be a potential indicator for identifying Chinese patients with T2DM at a higher risk of DKD and its distinct phenotypes.

Growing evidence has implicated the role of chronic inflammation andoxidative stress in the pathogenesis of DKD (49, 50), and inflammation and oxidative stress may be considered as a hub of the different pathogenic pathways that contribute to DKD (51). The imbalance of of several pro-and anti-inflammatory cytokines, such as tumor necrosis factor alpha (TNF-α), interleukin 6 (IL-6), C reactive protein (CRP), neutrophils, lymphocytes, and platelet cells, and pro- and anti-oxidants, such as superoxide dismutase (SOD), malondialdehyde (MDA), and 8-OHdG-8-hydroxy-2′-deoxyguanosine (8-OHdG) have previously been reported to be related to the development of DKD (4, 5, 49, 52, 53). Hb, an iron-containing protein in blood, may serve as a nitricoxide scavenger, and its ability of Hb to bind the main low-molecular-weight thiol of the cell glutathione, both covalently and noncovalently, is not only an important part of the antioxidant protection of red blood cells, but also affects its affinity for oxygen in both cases (54, 55). It has been reported that decreased Hb was correlated with a higher incidence of rapid renal function decline, and could predict the development and progression of DKD (56).

Bilirubin, a byproduct of normal Hb breakdown that plays an important physiologic role as a strong antioxidant and anti-inflammatory molecule through efficiently scavenging of peroxyl radicals and suppression of oxidation, inhibiting platelets, and regulating immunity (57), has been demonstrated to be involved in the development and progression of DKD (58). The present study provided evidence that inflammation and oxidative stress correlated with DKD and its distinct phenotypes, since we found that T2DM patients with DKD had significantly higher neutrophil count, and lower lymphocyte count, TBIL, and Hb than those with non-DKD, and neutrophil count was further increased and lymphocyte count, TBIL, and Hb were further decreased in T2DM patients with DKD-non-Alb and DKD stage 3 Alb compared to those with DKD stages 1–2 Alb, and TBIL and Hb were independently significantly associated with the presence of DKD. In addition, we demonstrated that SII was positively associated with neutrophil and PLT count, and inversely correlated with lymphocyte count, TBIL, and Hb, which was consistent with previous studies that reported that the subjects with higher quartile of SII have significantly higher neutrophil count, PLT count, and lower lymphocyte count, Hb and TBIL than those with lower quartiles, and SII levels were positively related to CRP, erythrocyte sedimentation rate, and 8-OHdG in patients with tumors, hypertension, diabetic foot infections, systemic lupus erythematosus, and alveolar hydatid disease (11, 22, 24, 59–62), suggesting that SII may be associated with chronic inflammation and oxidative stress, and chronic inflammation and oxidative stress might at least partially mediate the potential relationship between SII and DKD; However, the mechanism of action needs to be further investigated.

Evidence to date has suggested that hyperglycaemia, hypertension, dyslipidaemia, and IR are important risk factors for DKD (49, 63). Hyperglycemia is a widely accepted modifiable risk factor for the initiation and promotion of DKD through triggering three cardinal and inter-related pathways including overproduction of ROS, activation of apoptotic pathway and initiation of autophagy, especially in people with poor glycemic control (64). Elevated blood pressure and dyslipidemia were also identified as other major modifiable risk factors associated with the development and progression of DKD in T2DM individuals (64). Some studies have found that antihypertensive and lipid-lowering therapy can reduce the risk of albuminuria, kidney function decline, and progression to ESRD (35, 65). It has been recognized that IR is closely interrelated with hyperglycaemia, hypertension, and dyslipidaemia, and can increase the hydrostatic pressure of the glomerulus and permeability of the renal vessels, leading to glomerular hyperfiltration and subsequently microalbuminuria and DKD (66, 67). The apoB/apoA ratio has been reported to be significantly associated with IR in certain population including Chinese population, independent of traditional risk factor (68). Chronic glycemic exposure (the degree and duration of plasma hyperglycemia), as reflected by GE index, is thought to be the important modifiable risk covariate for diabetic complications (69). Our study provided further evidence that supported the potential role of hyperglycemia, hypertension, dyslipidaemia, and IR in the pathogenesis of CKD, since we found that the subjects with DKD had significantly higher SBP, MAP, TG, apoB/A, METS-IR, GE index, prevalence of hypertension, and dyslipidaemia, and lower HDL-C and apoA, and GE index, apoB, apoA, hypertension, and METS-IR, a novel inexpensive and reliable surrogate indicator of IR (34), remained independently significantly associated with the presence of DKD after adjustment for confounding factors. Moreover, partial correlation analysis controlling for sex, age, BMI, and duration of diabetes demonstrated that SII was positively associated with apoB/A, FBG, PBG, HbA1c, GE index, poor glycaemic control, METS-IR, and inversely correlated with TC and HDL-C, suggesting that SII might be correlated with metabolic disorders, especially IR, hyperglycemia, and dyslipidaemia, and metabolic disorders might at least partially mediate the relationship between SII and DKD. Our findings were consistent with previous studies that reported the potential relationship between SII and metabolic disorders (70–79). Some studies have demonstrated that the subjects with the highest quartile of SII have significantly higher homeostatic model assessment index of IR (HOMA-IR) than those with the lower quartiles in perimenopausal and postmenopausal women (70, 71), and the U.S. general population (72), and SII was positively associated with HOMA-IR in children with obesity (15). Furthermore, Yang and colleagues also found that CAD patients after coronary intervention with SII ≥ 694.3 had significantly higher glucose levels and rate of diabetes, and lower lipid profiles than those with SII < 694.3 in Taiwan (73). Similar results were reported in 9107 critically ill patients with HF from the Medical Information Mart for Intensive Care III (MIMIC III) database (74). SII was also considerably higher in patients with low HDL-C in rural areas from the Northeast China Rural Cardiovascular Health Study (NCRCHS) (17). However, some investigations have suggested significant opposite or no association between SII and dyslipidaemia, lipid profiles, and diabetes and its related indexes (FBG, HbA1c) in patients with different pathogenic conditions, such as hypertension, ASCVD, acute coronary syndrome patients with CKD, myocardial infarction, HF, and acute ischemic stroke (74–79). The discrepancies between the above-mentioned studies and ours may be due to the differences in study design and population characteristics, diabetic duration, races, regions, dietary habits, sample size, statistical methods, diagnostic methods for Dyslipidaemia and diabetes, and confounding factors adjusted. Large-scale and multi-center longitudinal studies are warranted to confirm the role of metabolic disorders in the relationship between SII and DKD.

Limitations of our study must be appreciated for an accurate interpretation of the data. First, we cannot infer causal associations between SII and DKD due to the cross-sectional design of this study. Further prospective studies are required to confirm this association. Second, we did not obtain information on educational level, dietary habits, lifestyle (smoking, drinking, and exercise), and consumption of various medicines, such as hypoglycemic agents, antihyperlipidemic drugs, anti-hypertension drugs, and antiplatelet drugs, which may have reduced our ability to explore other risks or protective factors. Third, participants in our study were from a single center and the great majority of them were middle-age or elderly, and hospitalized for relative poor glycemic control, its generalizability should be verified by involving outpatients or community patients in the future. Fourth, DKD-non-Alb subgroup and DKD stage 3 Alb subgroup were combined into a group called DKD-non-Alb+DKD stage 3 Alb group due to limited sample sizes of DKD-non-Alb subgroup. Therefore, prospective studies with larger sample size of DKD and each distinct phenotypes of DKD are still required to clarify the associations between SII and DKD and its distinct phenotypes. Last, the lack of classical inflammation and oxidative stress markers, such as TNF-α, IL-6, CRP, SOD, MDA, and 8-OHdG, make it difficult to further explore the association mechanism of SII and inflammation and oxidative stress in T2DM patients with DKD. Despite these limitations, our analyses also have some noteworthy strength. A key finding was that our study was the first study to assess the association of SII with DKD and its distinct phenotypes in Chinese patients with T2DM, which may provide additional information to identify those at risk for DKD and its distinct phenotypes, and thereby potentially institute earlier therapies. In addition, we recruited a relatively large clinical sample of patients with T2DM and performed this study with a comprehensive and standardized clinical assessment protocol, which can raise the reliability of our findings.

In conclusion, our data delineate that T2DM patients with DKD had significantly higher SII levels, and its levels were gradually increased moving from the non-DKD group to the DKD-non-Alb+DKD stage 3 Alb group. Moreover, SII was independently significantly associated with the presence of DKD and its distinct phenotypes after adjustment for confounding factors. These findings indicate that SII could be potentially used as an easy biomarker to identify those patients at high risk for DKD and its distinct phenotypes that further can help in choosing effective treatment options to delay the development and progression of DKD. However, further research is needed to perform for exploring their exact underlying mechanisms between SII and DKD in Chinese adults with T2DM.

## Data availability statement

The original contributions presented in the study are included in the article/**Supplementary Material**. Further inquiries can be directed to the corresponding author.

## Ethics statement

The studies involving humans were approved by the Affiliated Hospital of Southwest Medical University. The studies were conducted in accordance with the local legislation and institutional requirements. The participants provided their written informed consent to participate in this study.

## Author contributions

PY: Conceptualization, Data curation, Formal analysis, Funding acquisition, Investigation, Methodology, Writing – original draft, Writing – review & editing. YY: Data curation, Formal analysis, Investigation, Writing – original draft. XZ: Data curation, Formal analysis, Investigation, Writing – original draft. YZ: Data curation, Formal analysis, Investigation, Writing – original draft. JL: Data curation, Formal Analysis, Investigation, Writing – original draft. ZW: Data curation, Formal analysis, Investigation, Writing – original draft. XD: Data curation, Project administration, Writing – original draft. XW: Data curation, Project administration, Writing – original draft. XC: Data curation, Project administration, Writing – original draft. SL: Data curation, Project administration, Writing – original draft. YX: Data curation, Project administration, Writing – original draft. QW: Data curation, Project administration, Writing – original draft.
