# Supplementary material for: Association of systemic immune-inflammation index with diabetic kidney disease in patients with type 2 diabetes: a cross-sectional study in Chinese population
Source: Front Endocrinol (Lausanne). 2024 Jan 4;14:1307692. doi: 10.3389/fendo.2023.1307692 (PMC10795757; doi:10.3389/fendo.2023.1307692)
Supplement: Supplementary file 1 [file Table_1.doc]

**Supplementary Table 1 SII levels and other clinical characteristics in T2DM patients with Non-DKD and DKD.**

| **Variables** | **Non-DKD** | **DKD** | ***P*-value** |
| --- | --- | --- | --- |
|  | **(n =859 )** | **(n =1063 )** |  |
| Male, n (%) | 457 (53.20) | 518 (48.73) | 0.051 |
| Age (years) | 57.84±10.93 | 63.05±11.05 | 0.000 |
| BMI (kg/m2) | 24.26±3.46 | 24.43±4.06 | 0.646 |
| Duration of diabetes (years) | 6.41±5.75 | 9.51±6.73 | 0.000 |
| Family history of diabetes, n (%) | 241 (28.06) | 259 (24.37) | 0.067 |
| Family history of hypertension, n (%) | 82 (9.55) | 108 (10.16) | 0.654 |
| Smoking, n (%) | 180 (20.95) | 227 (21.35) | 0.831 |
| Drinking, n (%) | 149 (17.35) | 168 (15.80) | 0.365 |
| SBP (mmHg) | 127.73±19.73 | 138.28±23.02 | 0.000 |
| DBP (mmHg) | 71.76±11.97 | 72.83±13.26 | 0.122 |
| MAP (mmHg) | 90.42±12.70 | 94.65±14.31 | 0.000 |
| PP (mmHg) | 55.98±16.99 | 65.47±20.02 | 0.000 |
| TC (mmol/L) | 4.75±1.22 | 4.78±1.51 | 0.415 |
| TG (mmol/L) | 2.21±2.40 | 2.36±2.62 | 0.035 |
| HDL-C (mmol/L) | 1.18±0.35 | 1.15±0.41 | 0.003 |
| LDL-C (mmol/L) | 2.74±0.92 | 2.73±1.11 | 0.133 |
| apoA (g/L) | 1.36±0.35 | 1.26±0.32 | 0.000 |
| apoB (g/L) | 0.88±0.25 | 0.91±0.35 | 0.334 |
| apoB/A | 0.69±0.25 | 0.76±0.35 | 0.000 |
| FBG (mmol/L) | 10.63±4.77 | 10.78±5.64 | 0.482 |
| PBG (mmol/L) | 15.89±5.20 | 15.99±5.06 | 0.634 |
| HbA1c (%) | 9.32±2.44 | 9.28±2.56 | 0.460 |
| GE index | 3.47±0.78 | 3.77±0.77 | 0.000 |
| METS-IR | 40.72±8.64 | 41.86±10.10 | 0.040 |
| Neutrophil (*10^9^ /L) | 4.18±1.89 | 5.13±2.76 | 0.000 |
| Lymphocyte (*10^9^ /L) | 1.71±0.67 | 1.53±0.60 | 0.000 |
| PLT (×10^9^ /L) | 194.47±64.27 | 206.79±86.12 | 0.064 |
| SII | 563.07±18.43 | 891.63±34.40 | 0.000 |
| ALT (U/L) | 25.37±24.25 | 22.33±24.12 | 0.000 |
| AST (U/L) | 22.38±16.68 | 23.30±24.40 | 0.913 |
| TBIL (μmol/L) | 13.21±5.77 | 11.21±5.66 | 0.000 |
| Hb (g/L) | 134.82±17.12 | 119.70±21.69 | 0.000 |
| Serum Cr (μmol/L) | 60.52±15.85 | 106.58±103.53 | 0.000 |
| eGFR (mL/min/1.73 m2) | 101.25±16.60 | 76.15±32.80 | 0.000 |
| Urinary ACR (mg/g) | 13.34±7.59 | 748.30±44.89 | 0.000 |
| Overweight/obesity, n (%) | 411 (47.85 ) | 502 (47.22 ) | 0.333 |
| Poor glycaemic control, n (%) | 707 (82.31) | 850 (79.96) | 0.198 |
| Hypertension, n (%) | 371 (43.19) | 734 (69.05) | 0.000 |
| Dyslipidaemia, n (%) | 578 (67.29) | 769 (72.34) | 0.017 |
| DR, n (%) | 69 (8.03) | 183 (17.22) | 0.000 |
| ASCVD, n (%) | 211 (24.56) | 430 (40.45) | 0.000 |

Data are mean ±SD. SD, standard deviation; DKD, Diabetic kidney disease;

BMI, body mass index; SBP, systolic blood pressure; DBP, diastolic blood

pressure; MAP, mean arterial pressure; PP, pulse pressure; TC, total cholesterol; TG, triglyceride; HDL-C, high-density lipoprotein cholesterol; LDL-C, low-

density lipoprotein cholesterol; apoA, apolipoprotein A; apoB, apolipoprotein B; apoB/A, apolipoprotein B-to-apolipoprotein A ratio; FBG, fasting blood glucose; PBG, 2 hour postprandial blood glucose; HbA1c, glycated hemoglobin A1c; GE index, glycemic exposure index; METS-IR, metabolic score for insulin

resistance; PLT, platelet; SII, systemic immune-infammation index; ALT,

alanine aminotransferase; AST, aspartate aminotransferase; TBIL, total bilirubin; Hb, hemoglobin; Cr, creatinine; eGFR, estimated glomerular filtration rate;

ACR, albumin- to-creatinine ratio; DR, diabetic retinopathy; ASCVD,

atherosclerotic cardiovascular disease. vs. non-DKD: ^*^*P*< 0.05, ^**^*P*< 0.01, vs. Alb DKD stages 1- 2: ^#^*P*< 0.05, ^##^*P*< 0.01.
